# A 37-year-old Nigerian woman with Apert syndrome – medical and psychosocial perspectives: a case report

**DOI:** 10.1186/s13256-018-1638-7

**Published:** 2018-05-13

**Authors:** M. A. Kana, T. S. Baduku, H. Bello-Manga, A. S. Baduku

**Affiliations:** 1 Department of Community Medicine, Kaduna State University, Kaduna, Nigeria; 2 Department of Radiology, Kaduna State University, Kaduna, Nigeria; 3 Department of Hematology, Kaduna State University, Kaduna, Nigeria; 4 Department of Sociology, Kaduna State University, Kaduna, Nigeria

**Keywords:** Apert syndrome, Adult, Psychosocial, Rehabilitation

## Abstract

**Background:**

Apert syndrome is a rare genetic disease that presents a diagnostic dilemma because of its similarity with other craniosynostosis syndromes. Currently, there is paucity of reports about adult patients in African medical literature. Therefore, this case report highlights medical and psychosocial problems associated with the disease in an adult woman who is resident in a resource-constrained setting.

**Case presentation:**

Our patient is a 37-year-old African woman. She had abnormal characteristics of the skull, face, and extremities that were detected at birth. She is clinically stable but moderately depressed as an adult. Mutation in fibroblast growth factor receptor 2 (Ser252Trp) was positive. Her physical deformities and the laboratory findings confirmed the diagnosis of Apert syndrome. She missed opportunities for vital interventions to limit the physical and psychosocial effects of the disease, especially during early growth and developmental period, mainly due to the inadequacy of the institutions offering medical and psychosocial support. As a child she did not complete formal education or acquire vocational skills even though intellectual disability was never established. During adulthood she became socially deprived owing to her physical features and educational handicap. Her lifelong dependency is an unfortunate social consequence starting with developmental challenges encountered during childhood and worsened by adult social maladjustment.

**Conclusions:**

Our patient does not have medically life-threatening features but was depressed. We recommend strengthening of institutions for early medical intervention and lifetime psychosocial support to limit physical and psychosocial effects of Apert syndrome among adult survivors in resource-limited settings.

## Background

Apert syndrome, also known as acrocephalosyndactyly type 1, is a rare genetic disease that is characterized by craniofacial deformities and malformations involving the extremities and central nervous system with intellectual disability in some cases [1, 2]. This disorder accounts for 4% of craniosynostosis syndromes and its genetic inheritance is autosomal dominant with the mutation of fibroblast growth factor receptors (FGFR2) on chromosome 10 [3]. Certainly, this mutation is diagnostic of Apert syndrome and there are no other mutations associated with the disease. Fundamentally, most cases are sporadic and the incidence in the general population is low and globally 1 in 65,000 children are born annually with Apert syndrome but only 1 in 160,000 survive infancy [1, 4]. The disorder also has a familial manifestation as has been observed in a Congolese male patient and his mother with variation in severity of presentation [1].

Worldwide, the first documented case of Apert syndrome was made in 1894, while the first recorded case of a Nigerian child was in 1982 [5, 6]. The disorder derived its name from French pediatrician Eugène Apert who published a series of nine cases in 1906 [6]. Children with this syndromic craniosynostosis have been reported to survive and manage relatively well in adulthood [7]. However, much of the information on this genetic disorder focuses on its medical aspects with less about intellectual, behavioral, and emotional functioning during adulthood [8]. Presently, there is limited documentation about adults with the disease in sub-Saharan Africa and this is the first adult case report from Nigeria, Africa’s most populous country of over 160 million people [9]. Therefore, in this case report we aimed to highlight medical and psychosocial problems associated with the disease in an adult African female resident in a developing country setting.

## Case presentation

**Fig. 1:**
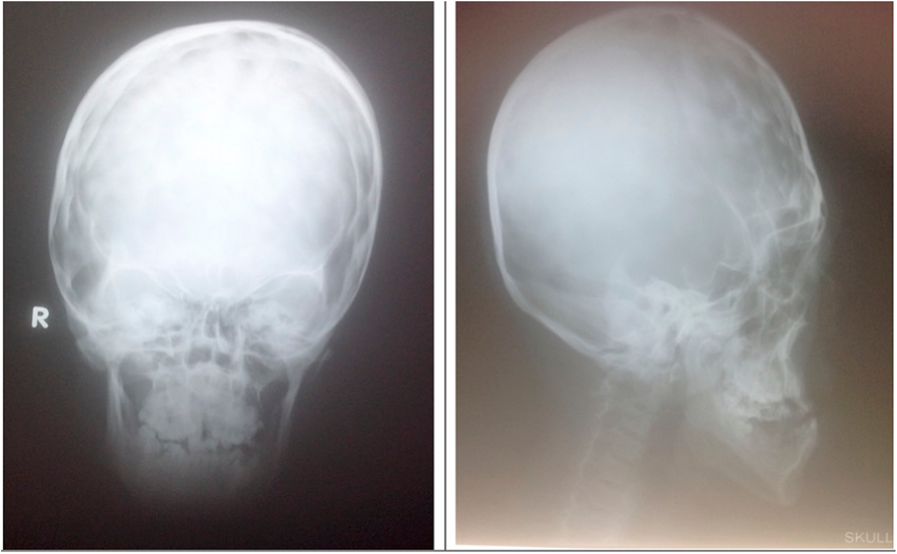
Anterior and lateral pictorial and X-ray views of the face

**Fig. 2:**
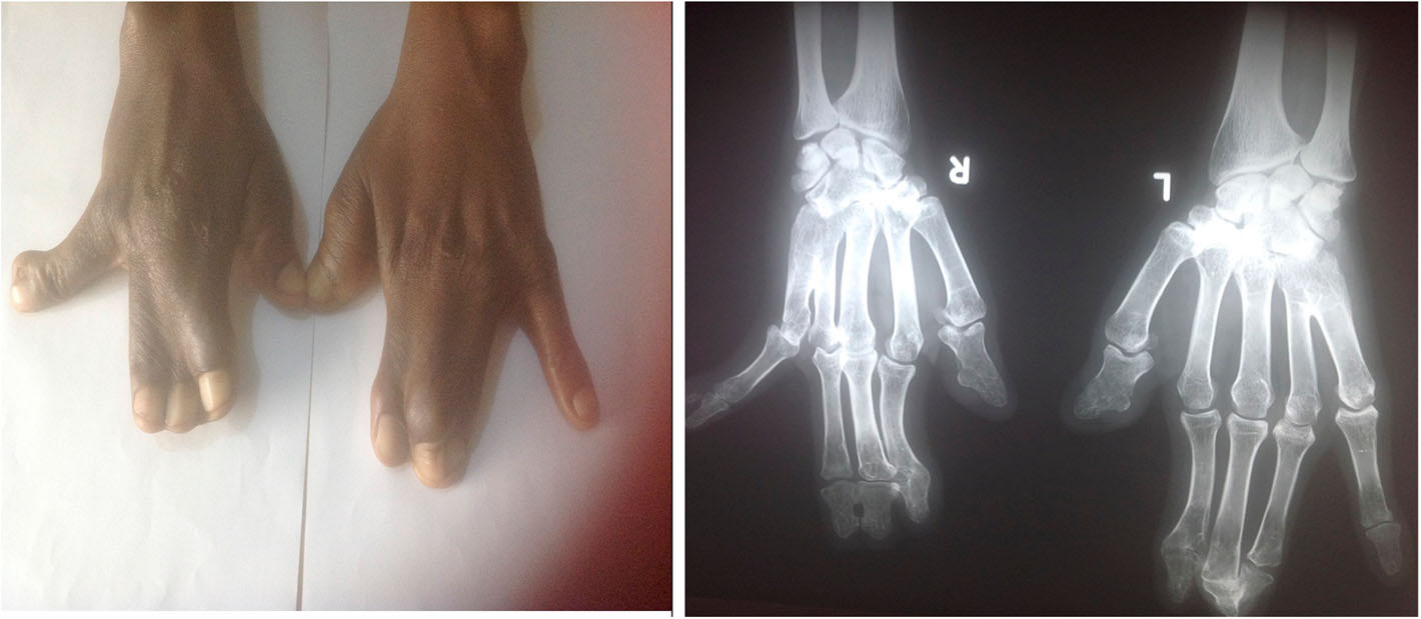
Picture and X-ray of the hands

**Fig. 3:**
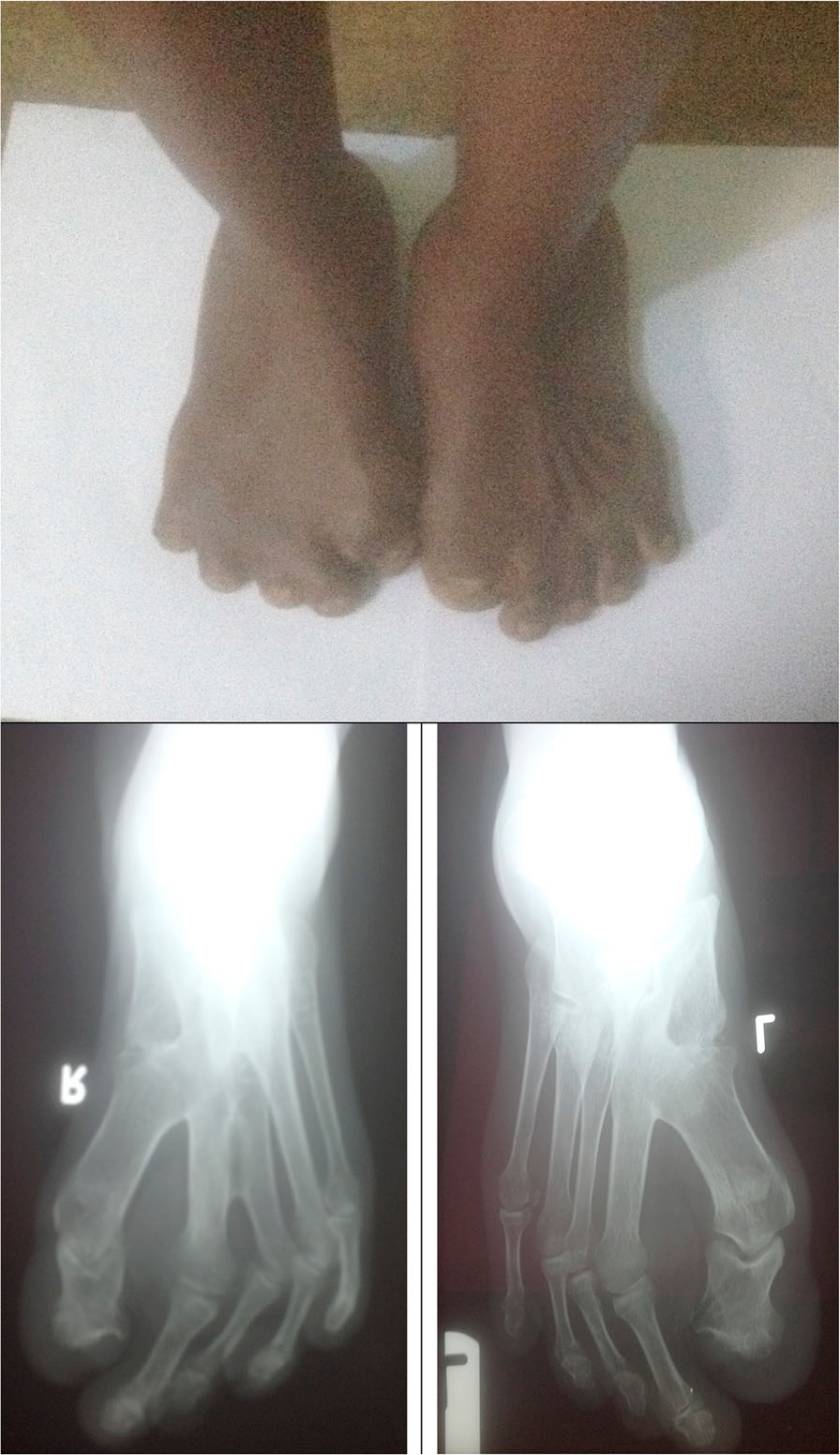
Picture and X-ray of the feet

Our patient is a 37-year-old African woman with Apert syndrome that was suspected at birth based on her physical deformities and later confirmed with a laboratory investigation. As an adult, she has had several medical consultations on account of illnesses like malaria but was never hospitalized. For this case report she had a comprehensive clinical evaluation, and radiological and laboratory investigations at the Barau Dikko Teaching Hospital (Kaduna, Northwestern Nigeria). A provisional diagnosis of Apert syndrome was made while a differential diagnosis of Crouzon syndrome was considered. A physical examination showed a young woman who was clinically stable, appeared older than her given age, physically and mentally alert, although very self-conscious of her physical appearance. Her cardiopulmonary and abdominal findings were within normal limits. She had an abnormal skull and facial characteristics with her eyes wide-set and bulging, which did not close well (Fig. 1). Her upper jaw was underdeveloped with crowded teeth and dental anarchy. There was no loss of teeth; palatal arch and nasal cavity were normal. Her second to fourth fingers were symmetrically webbed and fused (Fig. 2), whereas her first toes were underdeveloped with fusion of the other four phalanges (Fig. 3).

The Patient Health Questionnaire-9 (PHQ-9) was used to assess her for depressive disorder [10]. A score of 11 placed her within the range for moderate depression, which aligns with her constant feeling of unhappiness and worry about her physical deformity. She denies any history of physical, sexual, or financial abuse by others. She is not intellectually disabled, has no history of alcohol or drug abuse, and does not require assistance to undertake activities of daily living. Her speech and language abilities were normal and sufficient for communication and learning.

Skull X-rays showed an elevated frontal bone and depressed midfacial bones (Fig. 1); the cranial vault showed a copper-beaten appearance. The first phalanx of her thumb is short and thick bilaterally while the second phalanges are underdeveloped (Fig. 2). There is fusion of the bases of the fourth and fifth metacarpals bilaterally while there is minimal bony fusion at the apices of the second to fourth proximal phalanges symmetrically. The bones of her feet indicated fusion at the bases of the first and second metatarsal bones bilaterally while there are medial angulations of the metatarsophalangeal joints (Fig. 3). Chest radiograph, brain computed tomography (CT) scan, electrocardiography, and echocardiography showed normal features. Hematological and blood chemistry parameters were within normal limits. Deoxyribonucleic acid (DNA) analysis done showed a mutation in the *FGFR2* (Ser252Trp*)* that was positive. This result of the genetic test complemented other clinical findings to confirm the diagnosis of Apert syndrome.

Her father and mother were 35-years old and 25-years old, respectively, at the time of her birth in 1979. She was a product of a normal term pregnancy that was characterized by regular prenatal care attendance and at the third trimester her mother had a history of leg swelling, which was not associated with other significant clinical complaints. The labor and delivery were at home, but our patient was immediately observed to have abnormal physical features affecting her face, hands, and feet. At 3 months of age, she was admitted for 12 weeks at Ahmadu Bello University Teaching Hospital (Kaduna, Northwestern Nigeria) for clinical evaluation, pre-surgery preparation, and surgical operation. The surgical plan was to correct deformities of her eyes, hands, and feet. However, the operation was not done due to an anesthetic complication that resulted in her lapsing into unconsciousness after which she was resuscitated. The procedure was rescheduled but the unfavorable experience of the first attempt discouraged her family from seeking further surgical intervention. She had normal developmental milestones in terms of dental maturation, speech, crawling, and walking as well as social interaction. Although as an adult she expressed desire for an improvement in her physical features, she is afraid of complications because of what her parents narrated about the earlier attempt.

She was enrolled into a public school as a child and coped well until it was terminated after 3 years. This was mainly due to constant teasing of her abnormal physical features by schoolmates. Eventually, she did not have any formal education or vocational training and took up street begging at 12 years. Later as an adult she intermittently resided in a shelter for disabled people where she felt normal among other persons with physical disabilities. The shelter is not well organized and funded to cater for their basic needs like food and health care. Hence, the disabled persons routinely beg on the street as a source of livelihood. It was in this shelter that she interacted with male disabled persons and got married three times. The first husband died from severe burns resulting from fire that gutted their home and the second marriage ended in divorce because the husband was incapable of meeting her basic needs. She could not recall her age at her first marriage or its duration or the interval between the end of her first marriage and the start of her second marriage. She was pregnant once during the second marriage, which ended in miscarriage. A third marriage was contracted approximately 3 months before our first interview with her in August 2015. The third marriage was short lived and ended in divorce due to disagreements. All her husbands were beggars characterized with various forms of physical deformity. She still desires to re-marry so that she can live an independent life.

She currently resides with her extended family where she feels accepted and not stigmatized. She affirmed that she related well with them and is happier at home. However, her relationship with her family was sometimes strained because of her refusal to stop street begging. Furthermore, the family expects and exerts pressure on her to get married because all her younger sisters were married. She is the eldest daughter among the 12 children (4 males and 8 females) in a polygamous setting. All the children were alive and none had her type or any other form of genetic disorder. Her siblings considered her a stubborn but motivated person who attains goals once she sets her sight on them. At the time of the interview, her father was a 71-year-old pensioner, while her 62-year-old mother had never been engaged in formal employment.

## Discussion

The case we presented was clinically stable and had physical deformities characterizing Apert syndrome, which was detected at birth but an anesthetic complication deprived her of the opportunity for early reconstructive surgery. She did not acquire any formal education or vocational skills even though intellectual disability was never established. Later as an adult, she became socially disadvantaged owing to her physical features and educational handicap. She commenced street begging early in life and might not be entirely motivated by deprivation since her family had disapproved of this practice. At different times she attempted forming an independent life but all three marriages were unsustainable with persons with disability who also needed care.

In general, the findings of our case tally with recognized social features of adult survivors of Apert syndrome [7]. The lifelong dependency is an unfortunate social consequence that starts with developmental challenges encountered during childhood and worsened by adult social maladjustment. Compared to their normal peers they have lower education, are less often married, have fewer friends, and a majority live with their parents [7]. Although children who have syndromic craniosynostosis have full-scale intelligence quotients (FSIQs) similar to the normative population, they are at increased risk for developing intellectual disability and social problems [8]; thus, underscoring the need for early and comprehensive rehabilitation so as to enhance their quality of life. Unfortunately, resource-limited settings lack organized and coordinated medical and psychosocial interventions to cater for cases or support families to cope with its challenges.

The effect of genetic diseases on the family has several dimensions that straddle the lifespan of the patient. It is known that the birth of a child with craniofacial disorders creates tremendous stress on the parents’ coping resources [11]. This situation can expand to affect the whole family as we have observed with the case in this study. Therefore, it is beneficial for patients to be referred to support organizations to receive individual and family support [2]. Support groups for parents of children with genetic disorders, like craniofacial syndromes, will provide them counseling opportunities to share and discuss parental challenges as is obtainable in developed countries.

This patient missed opportunities for vital interventions to limit the physical and psychosocial effects of the disease, especially during her early growth and developmental period, mainly due to the inadequacy of the institutions offering medical and psychosocial support. The lifetime prognosis of Apert syndrome is variable depending on the severity of its manifestation as well as the quality and timing of treatment [2]. Genetic counseling and screening could assist diagnosis in the prenatal period; if patients are followed closely for developmental delays, early intervention therapies can be applied when required. As the child attains school age a psychological evaluation can be scheduled to detect and institute an intervention plan targeting cognitive/academic skills, language, and/or social-emotional development problems [2]. Management of physical deformities is preferably started during childhood, but if this is not done then later orthodontic and surgical treatments can be offered to an adult patient like our case [12].

## Conclusions

An adult with Apert syndrome in a developing country faces psychosocial disadvantages that worsen the physical disability associated with the disease. Unfortunately, developing countries like Nigeria lack organized genetic counseling, early diagnostic services, and comprehensive rehabilitation services. We recommend development and implementation of relevant policies, strengthening institutions, and enhancing interventions that will boost public awareness about the disease and provide sustainable lifetime care and support.
